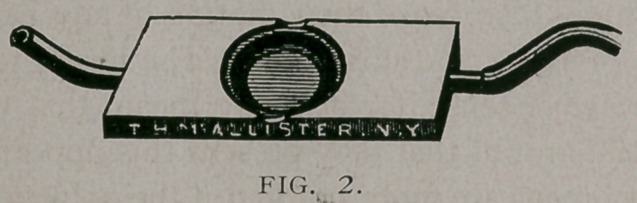# The Blood

**Published:** 1880-04

**Authors:** T. E. Satterthwaite

**Affiliations:** President of the Pathological Society of New York, etc.


THE ARCHIVES

OF

COMPARATIVE MEDICINE AND SURGERY.

VOL. I. APRIL, 1880. No. 2.

### Original Articles, Selections, and Translations.

### ART. VI—THE BLOOD.

BY T. E. SATTERTHWAITE, M. D.,

PRESIDENT OF THE PATHOLOGICAL SOCIETY OF NEW YORK, ETC.

THE blood of man and vertebrates in general consists of a clear fluid, the *liquor sanguinis* or *plasma*, in which are pretty evenly distributed a great number of corpuscles. Two principal varieties are met with, the red and the colorless, or white. The former preponderate very greatly, existing normally in the proportion of 600 or even 1,200 to one. They give to the liquid its characteristic red appearance. In fresh blood no solid matter except the corpuscles is to be seen under the microscope; the same is true in coagulated blood where the amount is large. When, however, a small quantity is allowed to dry, fibrin may be deposited under the form of very delicate filaments, which cross each other at varying angles. In one hundred volumes of blood there are thirty-six volumes of corpuscles and sixty-four of plasma. The *red corpuscles* in man and mammals, with very few exceptions, are bi-concave bodies, circular in outline; in birds, amphibia, and almost all fishes they are also bi-concave, or hollowed out at the centre, but have an elliptical contour. In the human species nuclei or central bodies appear at a very early period of life, but subsequently become invisible; in birds, amphibia and fishes a distinct rounded prominence is seen at the centre, when the corpuscle is turned so that its edge meets the eye of the observer. This prominence corresponds to the ordinary nucleus of other elementary corpuscles or cells. The rounded edges of these bodies and their excavated centres have induced writers to compare them to the little cakes, known as lady-fingers. It is obvious that when the corpuscle is seen flatwise the horizontal plane of the periphery and of the surface of the centre are different, and accordingly can neither of them be precisely in focus at the same moment. When one is dark, the other is bright; when one is sharp and distinct, the other is blurred.

Similar conditions cause, in all probability, the double contour that is often observed, although this very appearance has been regarded by some writers as good evidence that the corpuscle is surrounded by a wall of material differing in its nature from the central substance. It is well known, however, that all bodies which have a rounded margin refract light more or less. The sharper the defining power of the optical combination, the less of the double contour is seen; on the other hand, the poorer in quality the lens and eye-piece, the more is seen of double borders.

When lenses have a very great magnifying power the phenomenon is apt to be very marked.

Improper appreciation of these optical properties of the microscope have misled observers, so that they have obtained false conceptions of this structure. Unfortunately even now we are hardly in a position to decide upon the real structure of the elementary corpuscles of the body, and a certain wide range is open for theories.

*Measurements of the Red Corpuscles in Man and Animals.*— The average diameter of the human red globule is still a matter of discussion. The faulty measurements of the older writers have led to some misconception on these points, and we have been obliged to give the matter new study. Welcker, who has long been an authority on the Continent, gave .00774 millim. as the average breadth in the male. He had also observed a minimum of .0045. Max Schultze has recorded a maximum measurement of .010 millim. The average thickness is placed at .0018 millim. (Frey). According to recent investigations by Hayem, a diameter of .0120 or .0140 millim. may be reached, or it may be diminished to .0022 millim. Elsberg gives the mean diameter of the red blood corpuscles at .0075 millim., agreeing very nearly with Welcker. He has also observed a minimum diameter of .00422 and a maximum of .01016 millim.

Yet the average of one hundred measurements may vary in different individuals, or in the same individual. These points are of value in medico-legal cases where it is sought to prove that the suspected liquid is or is not human. In the blood of the pup, for example (the size of the corpuscle in the dog being very nearly that given for man), a recent observer found that the average diameter of fifty corpuscles varied only two-millionths of an inch from a like average of fifty corpuscles taken from his own blood. And again, in another instance, that forty from the pup varied only seven-millionths of an inch from a similar number in his own blood (Woodward).

As will be seen by referring to the table which follows, other of the domestic animals, as the cat and rabbit, have corpuscles whose average size is very nearly that of man, so that, with the variations to which all are subject, the differential diagnosis in a medico-legal point of view is likely to be difficult if not impossible. When the corpuscles have been dried, as in most of the instances where microscopical evidence is wanted, the shrinkage of the disks adds still further uncertainty to the result. Where corpuscles are elliptical, as in birds, there is less difficulty.

The following table gives a series of measurements, mostly by Welcker. In some the form is round, in others elliptical :

| | Average Diam. | Min. Diam. | Max. Diam. |
|---|---|---|---|
| Dog . . . . . . . . . . | .0073 | .0065 | —82 millim. |
| Cat. . . . . . . . . . | .0065 | .0058 | —74 |
| Rabbit . . . . . . . . . | .0069 | .0062 | —80 |
| Sheep . . . . . . . | .0050 | .0038 | —56 |
| Goat (old) . . . . . . | .0041 | .0036 | —46 |

| | Average Diam. | Min. Diam. | Max. Diam. |
|---|---|---|---|
| Goat (8 days old) . . . | .0054 | .0039 | —-66 millim. |
| Moschus Jaranicus. . . . | .0025 | .0022 | —-30 |
| Elephant.. . . . . . . | .0094 | .0084 | —106 |
| Pigeon (old).... . . . | .0147 | .0132 | —160 |
| " (fledgling) . . . | .0126 | .0116 | —140 |
| Chicken . . . . . . . | .0121 | .0104 | —132 |
| Duck . . . . . . . . . . | .0129 | .0118 | —140 |
| Vespertilio n., . . . . . | .0061 | .0054 | —-66 |
| Triton Cristatus... . . . | .0293 | .0259 | —327 |
| Salamandra . . . . . . | .0378 | .0302 | —415 |
| Cryptobranchus : | | | |
| Japonicus . . . . . . . . | .0512 | .0460 | —579 |
| Lepidosiren annectens | .0410 | .0360 | —440 |

| | Average Length. | Average Breadth. |
|---|---|---|
| Proteus auguineus . . . . . . . . . . . . | .058 millim. | .034 millim. |
| Amphiuna tridactylum (Schmidt, &c).... | .075 millim. | .047 |

*The Number of the Red Globules.*—It has been computed that the blood of an ordinary man contains 5,000,000 red corpuscles in each cubic centimeter. In anæmia the number is said to be rarely reduced below 3,000,000. In fair states of physical health, the amount may reach 6,000,000. Under ordinary circumstances 4,500,000 is thought to argue a fair bodily condition (Keyes). Cases are on record, however, where the number has been reduced to 800,000, a condition called by Hayem *aglobulie intense ;* in an instance, however, of what is termed *aglobulie extreme*, he has recorded a minimum of 450,000.

*The Blood Corpuscles in an Indifferent Fluid.*—One of the best means of getting a knowledge of the corpuscular elements of the body, is by a study of the blood in one of the lower animals. Specially adapted for this purpose is the blood of a rog or newt. The blood elements of these little animals are comparatively large, so that it is easy to study them with lenses of low power. At first we should observe them in a menstruum similar to the liquor sanguinis or plasma of the blood. For this purpose it is common to take a drop of aqueous humor from a frog. Add to it a drop of blood from the animal to be examined; stir them together, and then mount upon a slide, covering the drop with the ordinary three-quarter inch circle. The aqueous humor has no other effect upon the corpuscles than the serum of the blood. The material of which they are made appears to be homogeneous, that is, it is made up of structureless protoplasm, and there is usually neither nucleus nor central body to be seen. If it be impossible to employ aqueous humor, an excellent substitute may be obtained in the fluid from a hydrocele or ovarian cyst. To six ounces of this substance twenty grains of finely pulverized iodine may be added. After prolonged agitation, the iodine will be dissolved, and the mixture thus prepared may be kept for a considerable time. By the same method the *leucocytes* or *white corpuscles* will be seen to advantage. They are much smaller than the red globules (in the frog)—the reverse of human blood, and there is great range in dimension; one observer (Klein) having described as many as thirty different varieties.

Their contents are granular, that is, they appear to contain molecules in the interior; in the newt's blood it is not uncommon to see dark points in constant oscillation—the *Brownian movement.*

Klein explains this phenomenon in the newt's corpuscle as being the movement of "the disintegrated network," and due to the imbibition of water. Acting upon this explanation, the motion in the mucous corpuscle of the human saliva would indicate death rather than life.

This oscillatory motion ceases as the fluid is withdrawn by evaporation. Another phenomenon can also be seen in the same specimen under favoring conditions, viz: the *amoeboid*, so called because it resembles the action of the amoeba, the little microscopic organism found in stagnant pools of water. As the observer watches the leucocytes he sees them putting out longer or shorter processes, and withdrawing them, moving about as they do so from place to place. These changes will continue longer, if a little oil or glycerine be painted about the edge of the cover to prevent evaporation, and if the specimen be mounted upon a heated slide. Both Brownian and amoeboid motions are

FIG. I, HEATED SLIDE.

*a* Slide. *b* Copper Plate. *c* Arm over which the spiral wire (*d*) is slipped usually confined to a limited number of the leucocytes, and the former is often seen in only a portion of the corpuscle. The heated slide consists of an ordinary glass slide, (Fig. I *a*,) upon which is fastened a thin copper plate (*b*) perforated in its centre, so as to allow space for the drop of blood which is to be examined. From the copper plate extends an arm (*c*) over which a copper wire (*e*), wound spirally, is slipped, which is heated by the flame of an alcohol lamp. The glass plate is kept warm in this way, and with it the drop of blood. In order to obtain the requisite amount of heat, and no more, it is customary to put a little bit of cocoa butter upon the corner of the slide. The butter melts at the temperature of the body, and when this point is reached, the lamp should be carried nearer or removed further away, so that the temperature will be maintained constantly at a uniform degree.

*Action of a Dilute Salt Solution.*—It is often difficult, and indeed impossible, to obtain aqueous humor or even an animal fluid such as has been described, and microscopists have accordingly made use of a substitute which can be prepared at any time and kept indefinitely. This is a solution of common salt in distilled water (1-400). Add a drop of fresh frog's blood to a drop of the salt solution, mix them well, and it will be seen that the delicate protoplasm of the red blood corpuscle, the most susceptible of change, is not altered in appearance, there being merely a change in form from the elliptical to the spherical.

This solution has been found in practice a very efficient substitute for serum, and it is therefore very generally used in examining fresh specimens, when it is important that no alterations be artificially produced in the material of which the corpuscle is made.

*Action of Distilled Water—Irrigation.*—The effect of water upon living corpuscles is also important to histologists and pathologists, as we are frequently in a position to see its results in studying animal tissue. Take a drop of frog's blood, add to it a drop of distilled water, and apply a cover. The nucleus or central body of the corpuscle will now come distinctly into view surrounded by a yellow border; the body of the corpuscle or peripheral part will at the same time become paler and larger. Add distilled water slowly, drop by drop, in the following way: Take a long strip of tissue or filter paper, about half the length of the slide and in breadth equal to half the diameter of the cover. Add water with an ordinary minim dropper close to the edge of the cover. The tissue paper will take up the excess of water and cause a stream to pass through the specimen. This is called *irrigation.* If the tissue paper be very thin and pushed a short distance under the edge of the cover, the solid particles in the fluid will be drawn to the edge of the paper where they will remain at rest, and can be observed at one's leisure.

Continued application of water will cause the corpuscles to swell up and eventually burst, or become so attenuated that they can scarcely be seen.

When water is slowly added to a drop of human blood, the corpuscles soon begin to lose their disk-like form and become spheroidal, or perhaps spherical. The coloring matter then often escapes, leaving them clear and entirely colorless. Such a condition is often seen in the human urine where the water of the liquid has been absorbed. The corpuscles then appear as colorless rings.

In the blood of a frog or newt, the body of the corpuscle is generally the first to imbibe water; later the nucleus, which at first is distinct. Sometimes the material of which the corpuscle is composed (haemoglobin) is gathered about the nucleus, sending off radiating prolongations to the periphery, while the imbibed fluid is stored in the intervening spaces.

*Action of Carbonic Acid Gas.*—This experiment requires a special apparatus. First of all it is essential to have a *moist chamber.* (Fig. 2.)

Take a small, flat bit of wood about 1½ inches wide, 3 inches long and ⅜ inch thick; make a square opening in the centre, sufficiently large to admit an ordinary ¾ inch cover-glass; this is to be pressed to the bottom and firmly fixed, thus making a shallow well with a glass bottom. Into this chamber are admitted, through side holes, glass tubes (one on each side), so that air or gases can be carried into the chamber. When in use, the chamber is kept moist by a drop of water, which is put in one corner of the well, while the specimen of blood to be examined is dropped upon a large glass cover, and the latter inverted over the mouth of the well. In determining the effect of carbonic acid gas upon animal life, we have merely to connect the gas chamber just described with a jar in which carbonic acid gas is generated. Fig. 2 illustrates a *gas* or *moist chamber* of the same general character, and devised by Dr. J. H. Hunt, of Brooklyn. Take a large gallon flask, fill it partly full of pulverized marble dust; connect it by means of a rubber tube, through a perforated stopper with a Wolff's bottle, which latter must be connected with the moist chamber. Now generate the carbonic acid gas in the flask, by pouring muriatic acid upon the marble dust. When the gas is being evolved it will be known by the ebullition in the Wolff's bottle. Now place the moist chamber upon the stage of the microscope. Take a drop of newt's blood (for it is more susceptible of change than frog's), dilute it with serum or an indifferent fluid, and mount it upon a glass cover, which invert over the well, first seeing that the edge of the cover is oiled, so that it will remain in place. Now connect the tube of the moist chamber with

FIG. 2.

the tube of the gas generator, and the carbonic acid gas will enter and pass through the chamber. The rapidity with which the current moves may be regulated by a spring clip. As soon as the gas enters, the central body or nucleus becomes distinctly visible, and is surrounded by a yellow halo; when, however, the gas is withdrawn and atmospheric air is admitted, these phenomena disappear. This central body, under such circumstances, has been called the *zooid*, and the corpuscle proper the *oikoid* (Bruecke).

A similar appearance may be produced by adding one drop of a two per cent. watery solution of tannin to a drop of human blood.

*Action of Acids Upon the Blood.*—Acetic acid is commonly used in observing the changes that are produced by an acid solution.

Take the ordinary dilute watery solution of acetic acid (1 per cent.) so much used in laboratories; add a drop of it to an equal amount of frog's blood. The red globules instantly exhibit nuclei. The colorless globules also cease their motion, if any has existed, and they become granular and shrivelled. The term granular is used merely in a relative sense, for it is not meant that the corpuscles are really granular, but that they present this appearance.

These phenomena are more marked if the solution is more concentrated. The red bodies, also, in such case, are apt to crack and split up. A good way of determining the proper strength for the ordinary acetic acid solution is to pour a little into an ordinary watch glass, and then add chemically pure acetic acid drop by drop until the solution is faintly acid to the taste.

*Action of Alkalies Upon the Blood.*—Take a drop of the newt's blood and mount it in a drop of serum or of salt solution. Then affixing a strip of bibulous paper in the way that has been described, add drop by drop a weak solution of liquor ammonia. A similar strip of paper, somewhat larger in size, upon the other side will cause a current and carry the corpuscles to the side of the field where the paper strip is largest, and there the corpuscles may be observed at rest, and the alterations effected by the alkali duly noted. It will be seen that after a little time the corpuscles, both red and colorless, will swell up and finally, after a time, if the alkali be in sufficient amount, disappear or become so attenuated as to be invisible. Sometimes they will burst and disappear, leaving the field evenly stained with a homogeneous glutinous-looking substance.

*Action of Electricity.*—It seems to make little difference, so far as the microscope is concerned, whether the continuous or the interrupted current is employed, as in either case the phenomena observed are the same in quality. Take bits of tin-foil and attach them to an ordinary glass slide, in such a way that they are just one-fifth inch distant from one another. The pieces of foil should be triangular in shape and have their pointed extremities turned to one another. The specimen should be a drop of newt's blood diluted with an equal amount of serum, both of which should be perfectly fresh and intimately mixed.

Placing a drop of this solution upon a cover-glass, it should be inverted and dropped upon the slide in such a way that it occupies an intermediate position between the bits of tin-foil. The ordinary stage clips of the microscope are then to be used to hold the slide firmly in position and press upon the tin-foil. The only remaining task is the attaching of conducting wires from the electrical instrument, one to one clip and one to the other. The bits of tin-foil are easily fastened to the slide; they have merely to be hammered out flat; they will then adhere by simple pressure. Sometimes it may be desirable to approximate the poles. In such cases it is necessary to use two fine bits of platinum wire. They should be flattened, and shaped like the figure S. Rest them upon the bits of tin-foil, opposite to one another and at the required distance apart. The cover-glass should press on them. Some little mechanical dexterity is required to get them in position, and they are apt after using to become polarized, so that the action upon the corpuscles is commenced before they are connected with the battery. The phenomena at the negative pole are those of an acid; of the positive, those of an alkali. At a distance from the line of the current, secondary changes occur of a less regular character.

Harting has devised an apparatus which is somewhat more elaborate, but in principle the same.

*Other Changes in the Red Corpuscles.*—If a drop of blood be taken from the finger, by pricking with a needle (the triangular or glover's is the best), it will be seen after a time that the peripheral portion becomes indented or *crenated*, as this change is called.

*Examination of the Circulation in the Web of a Frog's Foot.*—Take a medium sized frog and curarize him by injecting beneath the skin, with an ordinary hypodermic syringe, two drops of a weak solution of curara (1-2000 in water) or a few minims of a fifty per cent. solution of chloral hydrate (Schaefer). After a few minutes the animal will be completely paralyzed, but the circulation will go on without alteration.

Envelop his body in a damp cloth and extend him upon a cork plate about a quarter inch thick and large enough to support the entire body. Make a small opening in the cork, and over it place the web of the frog's foot, fastening the latter by ordinary pins.

The circulation may in this way be studied at one's leisure. The red and white blood corpuscles are seen in the arteries, veins, and capillaries. While the red bodies pass rapidly through the central portions of the vessels, the white creep slowly along the walls, altering their shape as they meet with any obstruction. Where, however, a small artery divides, it will sometimes be seen that the corpuscles, especially the red, are caught at the bifurcation; part bending to go down one branch, and part down the other; taking, in fact, the shape of a saddle-bag. Such a phenomenon exhibits the elastic and distensile properties of the corpuscle. Apply an irritant, such as a weak solution of nitrate of silver, and after prolonged and careful watching, the gradual exit of both white and red corpuscles (diapedesis) may be seen. This procedure requires extreme patience and a co-operation of peculiarly favoring conditions, which are not likely to favor a student who is just beginning the study of microscopy.

*Internal Structure of the Red Corpuscles.*—As yet the intimate structure of blood corpuscles is a matter little understood, though an abundance of theories are rife about it. Klein maintains that these corpuscles, in common with others in the body, are traversed by an intracellular network. In the red corpuscles of the newt, especially, he says there is a network of fibrils, with an inter-fibrillar hyaline ground substance, both together forming the so-called *stroma*. The nucleus contains a network of fibrils in connection with the network of the corpuscle proper; the haemoglobin, a colored fluid, is contained in the substance of the meshes of the network of the corpuscle proper. Drs. Cutter, of Boston, and Heitzmann, of this city, believe that there is also an intracellular network. The former regards it as due to the mycelium of a parasitic growth.

Real granules are often present, as may be proved by adding water in large quantity. They will then often burst, and the granules will be scattered throughout the field.

If finely ground vermillion is sprinkled in the liquid, some of the white corpuscles will take them up, often without losing their amoeboid character, and then eject them after a longer or shorter sojourn.

According to Boettcher, the human red blood corpuscle has a nucleus. He exhibits it in the following way: Taking a saturated solution of corosive sublimate in alcohol (96°), he diffuses about fifty volumes with one of blood. The corpuscles are deprived of their haematine, but at the same time are preserved. The mixture is frequently agitated, and in about twenty-four hours the corpuscles are allowed to subside, when the superincumbent fluid is poured off and alcohol added.

By further agitation for twenty-four hours they are thoroughly washed, and then settle at the bottom of the vessel.

Prof. Boettcher claims in this way to have found three classes of red corpuscles. The first are homogeneous and shiny throughout; the second are clear externally, but granular within; the third variety exhibit a nucleus and nucleolus.

Dr. Elsberg, of this city, also states that he finds a reticular appearance after using a solution of the bi-chromate of potash (30 per cent. to 50 per cent. of a saturated solution in water).

In early fœtal life all the corpuscles are colorless (Klein). According to Balfour and Foster, both colored and colorless corpuscles, at least in the chick, are developed from solid sprouts of protoplasm, derived from the middle germinal layer. Later the red ones make their appearance, and for a time are nucleated. The investigations of Neumann and Bizzozero, showing that the red corpuscles in the medulla of bones are also nucleated, favors the theory that the bone tissue is concerned in the transformation.

The personal observations of the author do not incline him to regard the network as definitely proved in fresh specimens, though it may and has previously been seen in corpuscles that have been exposed to chemical reagents.

*White or Colorless Blood Corpuscles.*—The white blood corpuscle is much larger, on an average, in the human species, than the red. It is round in form, and is estimated as varying between .0077 and .0120 mm. The average is .0091 mm. (Frey). In contour they are more or less rough, and exhibit processes. In some of these corpuscles the nucleus is distinct. When quite fresh, no nucleus is usually seen. If the eye of the observer can watch the corpuscle when it is upon a heated stage and under suitable conditions, the division of the cell may be seen.

According to Recklinghausen, the colorless corpuscle may be generated from the red corpuscle. According to Bizzozero and Neumann, a transitional corpuscle is met with in the medulla of bones. Neither of the two varieties of corpuscles, the red or the white, have a cell wall or outer investing membrane that can be demonstrated. It is not unlikely, however, that the outer layer of protoplasm has greater density than the more internal portions.

According to Dr. Richard Norris, there is, in mammals, a third corpuscular element which is usually invisible and of the same size as the red ones. Some doubt is thrown upon the alleged discovery by the fact that the method he employs is likely to produce artificial appearances, and therefore leads to the supposition that they are merely red corpuscles decolorized.

*Mode of Counting the Blood Corpuscles.*—Thanks to the instruments of Malassez, Hayem and Nachet and Gowers, we are in a position to count the red blood corpuscles with a fair degree of accuracy.

The methods are somewhat different, but are not difficult to understand. According to Malassez's, a given quantity of blood is mixed with one hundred parts of a solution of the sulphate of soda (ten per cent.). "Then a little of the mixture of blood and sulphate of soda is transferred to a very fine flattened capillary tube, the capacity of a given length of which is ascertained previously, and marked on the slide to which the tube is fixed." "On the capillary tube a length of four hundred micromillimeters* represents the 1–169.8 part of a cubic millimeter of the mixture. The counting is performed with the aid of a squared ocular micrometer, the microscope having been so arranged by observations of a stage micrometer, that the side of the square shall have the value of one of the lengths (four hundred micromillimeters for example) marked on the slide. The result of the counting gives the number of corpuscles in a known quantity (169.8 cubic millim.) of the mixture, and the number in the same volume of blood can readily be deduced" (Schaefer).

A plan more generally adopted is that of Hayem and Nachet. A capillary pipette is registered to contain two, two and a half and five cubic millim. of blood. When the finger has been punctured with a needle, the drop is sucked in through the point of the capillary tube. The larger pipette is registered so as to contain 200, 250, or 500 cubic millimeters.

Supposing that it is desirable to dilute the blood one hundred times, which is the usual dilution, blood is sucked into the capillary pipette until it reaches the point 2, and then the diluent is drawn into the larger pipette until it reaches the point 200. The two liquids are then mixed in the glass vessel and stirred with the rod. A rubber tube in the mouth makes the process of suction easier.

Now a slide is used, having a glass ring one-fifth millim. in depth, cemented on its upper surface. A drop of the mixture (but not enough to fill the ring so formed) is placed in the middle of the ring, and a perfectly flat covering-glass is so laid on that the drop touches and adheres to it without reaching the sides of the cell. The slide is placed on the microscope, and as soon as

---

* One one-thousandth of a millimeter.

the corpuscles have settled to the bottom of the drop, the number in a definite area is counted. If the area chosen is one-fifth of a millimeter square, this will give the number contained in one-fifth cubic millim. Multiply by twenty-five to get the cubic contents, and by one hundred (the times the blood was diluted) the result will be the number of corpsucles in a cubic millim. of blood. It is found most convenient to do the counting by means of the eye-piece micrometer, divided off into squares, each square being so placed with a suitable adaptation of lens and draw-tube, that the side of the square corresponds to one-fifth millim. measured on the slide. Having once made this measurement, and noted the depth to which the draw-tube should be pushed and the number of the lens and eyepiece, the examination can always be conducted with this combination, and thus much time be saved.

Rogers, of Boston, rules the micrometers very well.

The plan of Hayem and Nachet is the one usually adopted. Supposing that the blood has been diluted one thousand times, and the drop has been placed upon the slide and it is found that there are thirty-five corpuscles in a square of the micrometer (one-fifth millim.). Then in the same proportion there will manifestly be one hundred and twenty-five times that number in a cubic millim., and one thousand times that sum, since the blood has been diluted one thousand times—$35 \times 125 \times 1,000 = 4,375.000$—a condition which indicates fairly good health.

Dr. J. G. Richardson, of Philadelphia, has devised a plan for facilitating the methods of determining the ratio of red to white blood corpuscles that promises to be of much importance. In Malassez's method, the white corpuscles are apt to stick to the capillary tube, and consequently may fail to reach the measuring tube; in Hayem and Nachet's the density of the fluid may cause the corpuscles to be hid behind one another. To obviate this, he takes a drop of blood, places it upon the slide, and then with another slide having smooth ground edges, inclined to an angle of forty-five degrees with the first slide, draws it across slide No. 1, so as to spread out the drop equally; allowing it to dry, it will remain permanently unaltered, and the corpuscles will be found to be spread out in a little layer and at pretty even distances apart.

They can then be easily counted with the ordinary squared eye-piece micrometer just described.

There is a good deal of difficulty in the practical working of the *haematimetre*, which, with its expense, has prevented it from getting into general use. Some of them have arbitrary markings on the pipettes, or they may have no proper directions accompanying them. Besides, it is not certain that we know as yet the average number of corpuscles in the blood, for some extraordinary observations that have recently been made, tend to show that we must be careful in assuming 5,000,000 as the average number in the cubic millimetre of the human blood, a statement which has already been made.

Hayem and Malassez give the number at four and a half to five millions. The origin of the colorless corpuscles is still a matter of controversy. It seems probable that they are largely furnished from the lymphatic glands, and Klein thinks they are also thrown off from the "germinating buds" of serous membranes.

*Blood Crystals.*—The pigment of the blood occurs mostly in an amorphous form, and is called *haematine*. The brownish-red needles found in extravasated blood are known as *Haematoidine*. Haemoglobine also occurs under a cystalline form, which, in most mammals, consists of rhombic plates.

## Figures and Tables

**Fig. I f1:**
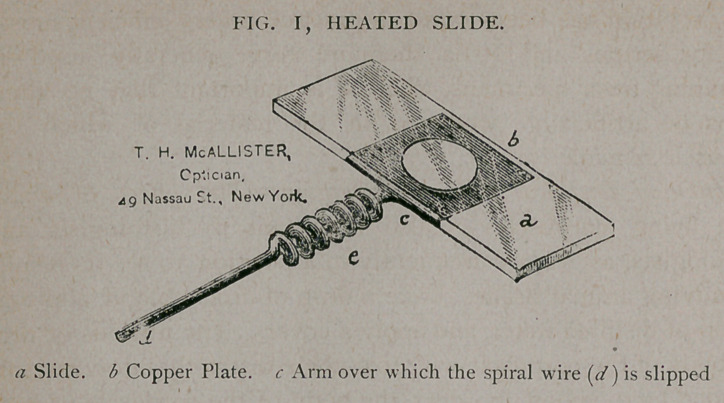


**Fig. 2. f2:**